# Expression and clinicopathological significance of miR-193a-3p and its potential target astrocyte elevated gene-1 in non-small lung cancer tissues

**DOI:** 10.1186/s12935-015-0227-8

**Published:** 2015-08-07

**Authors:** Fanghui Ren, Hua Ding, Suning Huang, Hanlin Wang, Mei Wu, Dianzhong Luo, Yiwu Dang, Lihua Yang, Gang Chen

**Affiliations:** Department of Pathology, First Affiliated Hospital of Guangxi Medical University, 6 Shuangyong Road, Nanning, 530021 Guangxi Zhuang Autonomous Region People’s Republic of China; Department of Radiotherapy, First Affiliated Hospital of Guangxi Medical University, 6 Shuangyong Road, Nanning, 530021 Guangxi Zhuang Autonomous Region People’s Republic of China; Department of Medical Oncology, First Affiliated Hospital of Guangxi Medical University, 6 Shuangyong Road, Nanning, 530021 Guangxi Zhuang Autonomous Region People’s Republic of China

**Keywords:** Non-small cell lung cancer, Carcinogenesis, MicroRNA-193a-3p, Metastasis, Astrocyte elevated gene-1

## Abstract

**Background:**

Aberrant expression of miR-193a-3p and astrocyte elevated gene-1 (AEG-1) have been revealed to be related to the tumorigenesis of various cancers, including non-small cell lung cancer (NSCLC). However, the significance of miR-193a-3p and its correlation with AEG-1 in NSCLC has not been explored. The purpose of this study was to evaluate the association between miR-193a-3p and AEG-1 and their relationship with the clinicopathological features in NSCLC patients.

**Methods:**

Via online in silico prediction, complementary sequences were found between miR-193a-3p and the 3′-untranslated region of AEG-1. Three independent cohorts were applied in the current study. Firstly, miR-193a-3p level was detected in 125 cases of NSCLC with quantitative real-time PCR (qRT-PCR). Secondly, AEG-1 protein level was evaluated in 339 cases of lung cancers with immunohistochemistry. Finally, the relationship between miR-193a-3p and AEG-1 protein expression was verified in another group with 65 cases of NSCLC.

**Results:**

The results showed that miR-193a-3p level was decreased in NSCLC tissues and significantly negatively related to tumor size (r = −0.277, P = 0.002), clinical TNM stage (r = −0.226, P = 0.011), lymph node metastasis (r = −0.186, P = 0.038), epidermal growth factor receptor (EGFR) protein level (r = −0.272, P = 0.041). On the contrary, AEG-1 protein expression was up-regulated in NSCLC and positively relative to tumor size (r = 0.240, P < 0.001), TNM stages (r = 0.164, P = 0.002) and lymph node metastasis (r = 0.232, P < 0.001) in NSCLC patients. In addition, miR-193a-3p was found to be inversely associated with AEG-1 protein expression in the third cohort (r = −0.564, P < 0.001).

**Conclusion:**

In conclusion, miR-193a-3p and AEG-1 might be responsible for the carcinogenesis and aggressiveness of NSCLC. AEG-1 has the potential to be one of the targeted genes of miR-193a-3p. However, future in vitro and in vivo experiments are needed to verify this hypothesis.

## Background

Lung cancer remains to be the leading cause of deaths associated with cancer worldwide [1]. Non-small-cell lung cancer (NSCLC), the predominant subtype, accounts for 75–80% of all lung cancer patients [2]. Despite advances have been developed in clinical and experimental oncology, the overall survival rate of 5 years for lung cancer is still unsatisfactory. However, there are no specific and sensitive biomarkers for diagnosis and prognosis prediction of NSCLC due to the complex mechanism underlying this malignancy. Therefore, it is urgent to explore the mechanisms and to discover new biomarkers for the prevention and prediction of NSCLC progression.

MicroRNAs (miRNAs), which are endogenous non-coding and evolutionarily conserved RNAs of about 19–24 nucleotides, can act as tumor suppressor genes or oncogenes [3]. In recent years, more and more studies have confirmed that miRNAs can regulate the processes of cancer metastasis, invasion, proliferation, apoptosis and prognosis [4–8]. MiR-193a is one of the miRNAs involved in carcinogenesis, which consists of miR-193a-3p and -5p. Researches have shown that miR-193a-3p plays essential roles in the multi-chemoresistance of bladder cancer through regulating PSEN1/HOX9/LOXL4 gene expression [9–11]. Moreover, decreased expression of miR-193a-3p was found to be correlated with metastasis, apoptosis and proliferation in breast cancer and ovary cancer [12, 13]. To date, four studies have reported that miR-193a-3p expression decreased in NSCLC tissues and hence, miR-193a-3p could be functioned as a tumor suppressor gene [14–17]. However, three of aforementioned four studies worked on NSCLC cell lines but not clinical samples [14–16], and only Yu et al. [17] studied the role of miR-193a-3p with human specimens, however, the patient size was small with 73 cases.

Astrocyte elevated gene-1 (AEG-1), also recognized as MTDH (metadherin) or Lyric (lysine-rich CEACAM1), was initially emerged as a HIV-1-inducible gene in human fetal astrocytes. Researches have demonstrated that AEG-1 contributes to several hallmarks of tumor progression, including metastasis, invasion and proliferation [18]. Furthermore, several studies also verified that increased expression of AEG-1 was intensely related to the carcinogenesis and progression of NSCLC [19–24]. Similar as other genes, AEG-1 can serve as targeted gene of several miRNAs, for instance, the correlations between miR-30c, -145, -375 and AEG-1 were proved to be responsible for the processes of NSCLC [24–26].

Based on the prediction in silico, complementary sequences were found between miR-193a-3p and the 3′-untranslated region of AEG-1. However, to our knowledge, no study has been reported on the association between miR-193a-3p and AEG-1 in NSCLC. Thus, this study was performed to investigate the relationship between expression of miR-193-3p and AEG-1 protein, as well as their clinical significance for NSCLC in three independent cohorts.

## Results

### Expression and clinicopathological significance of miR-193a-3p in NSCLC

**Fig. 1:**
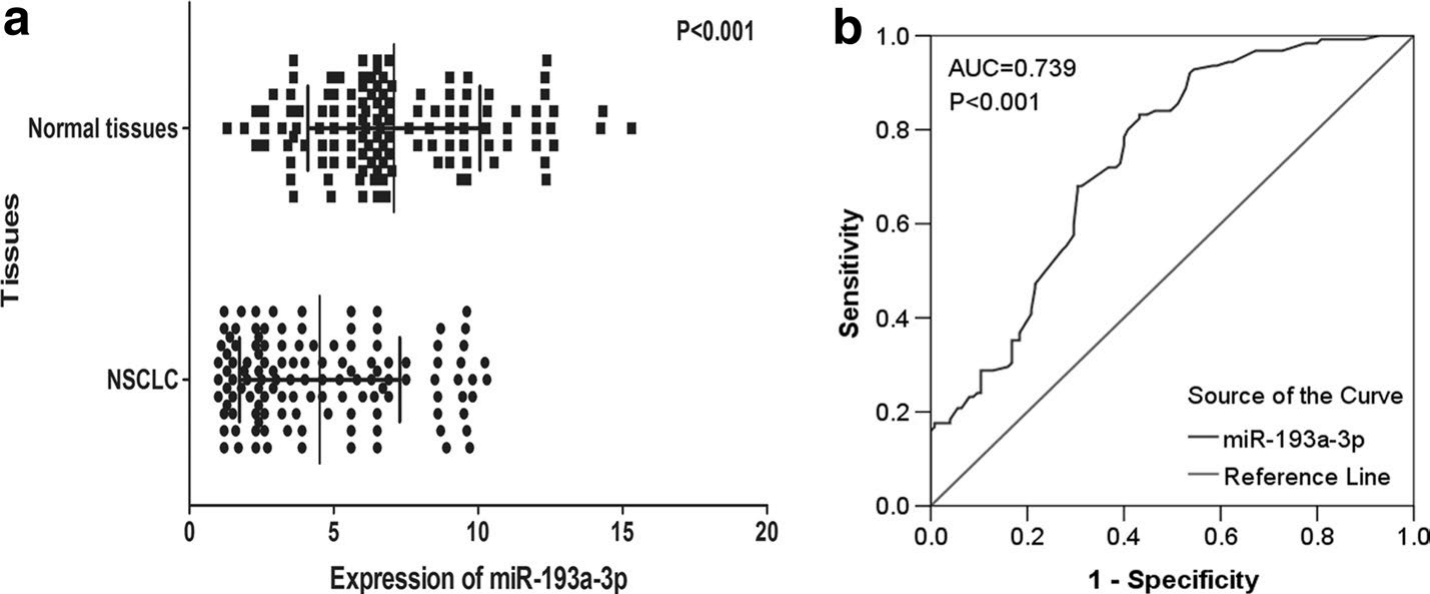
Expression of miR-193a-3p in adjacent non-cancerous lung and NSCLC tissues. Quantitative real-time PCR was performed to evaluate the expression of miR-193a-3p. **a** The difference of miR-193a-3p expression between adjacent non-cancerous lung and NSCLCC tissues. ***P < 0.001. **b** ROC curve of miR-193a-3p expression to distinguish NSCLC from non-cancerous lung. The area under curve (AUC) of miR-193a-3p was 0.739 (95% CI 0.678–0.800, P < 0.001), at a cut-off value of 0.4.* Error bars* represented SD. Significance of difference between two groups was analyzed by Student’s t test was performed to calculate the difference between two groups (**a** and **b**).

To measure the expression of miR-193a-3p, we performed qRT-PCR in 125 pairs of NSCLC tissues and matched non-tumorous tissues. The expression level of miR-193a-3p was 4.5076 ± 2.7700 in NSCLC tissues, which was significantly lower than that of normal tissues (7.0791 ± 2.9787, P < 0.001, Table 1; Fig. 1a). Moreover, as shown in Fig. 1b, the area under the curve from ROC of the miR-193a-3p was 0.739 (95% CI 0.678–0.800, P < 0.001). At a cut-off value of 0.4 estimated by the median expression of miR-193a-3p in all lung tissues, including NSCLC and normal lung, and the sensitivity and specificity were 0.3832 and 0.8500, respectively.

**Table 1:** The relationship between microRNA-193a and clinicopathological parameters in NSCLC

Clinicopathological feature	N	MiRNA-193a relevant expression (2^−ΔCq^)		
		Mean ± SD	t	P value
Tissue				
NSCLC	125	4.5076 ± 2.7700	−7.068	<0.001
Adjacent non-cancerous lung	125	7.0791 ± 2.9787		
Age (years)				
<60	57	4.3444 ± 2.4196	−0.602	0.549
≥60	68	4.6444 ± 3.0439		
Gender				
Male	75	4.3977 ± 2.6627	−0.542	0.589
Female	50	4.6724 ± 2.9433		
Smoke				
No	38	4.2087 ± 2.3248	0.461	0.646
Yes	30	3.9533 ± 2.1905		
Tumor size (cm)				
≤3	60	5.3203 ± 2.8395	3.273	0.001
>3	65	3.7574 ± 2.4984		
Lymph node metastasis				
No	56	5.0861 ± 2.8002	2.133	0.035
Yes	69	4.0381 ± 2.6739		
Vascular invasion				
No	90	4.3372 ± 2.6727	−1.104	0.272
Yes	35	4.9457 ± 3.0016		
TNM				
I–II	54	5.3269 ± 3.0154	2.885	0.005
III–IV	71	3.8845 ± 2.4074		
Pathological grading				
I	17	3.0353 ± 1.9371	F = 3.271^a^	0.041
II	78	4.8827 ± 2.7938		
III	30	4.3667 ± 2.8883		
Histological classification				
Adenocarcinoma	101	4.4685 ± 2.8159	0.276	0.759
Squamous carcinoma	23	4.5926 ± 2.6476		
Large cell carcinoma	1	6.5000		
EGFR amplification				
No	39	4.4905 ± 2.2605	1.501	0.139
Yes	18	3.4889 ± 2.5128		
EGFR protein expression				
Low	40	4.6058 ± 2.4525	2.181	0.034
High	17	3.1588 ± 1.8426		
EGFR mutation				
Wild type	44	4.1030 ± 2.2729	−0.415	0.680
Mutation	13	4.4154 ± 2.7501		

*NSCLC* non-small-cell lung cancer, *N* number, *SD* standard deviation, *TNM* tumor node metastasis, *EGFR* epidermal growth factor receptor.

Pathological grading I vs. II: P = 0.012, I vs. III: P = 0.110, II vs. III: P = 0.379.

^a^ANOVO test was performed.

**Fig. 2:**
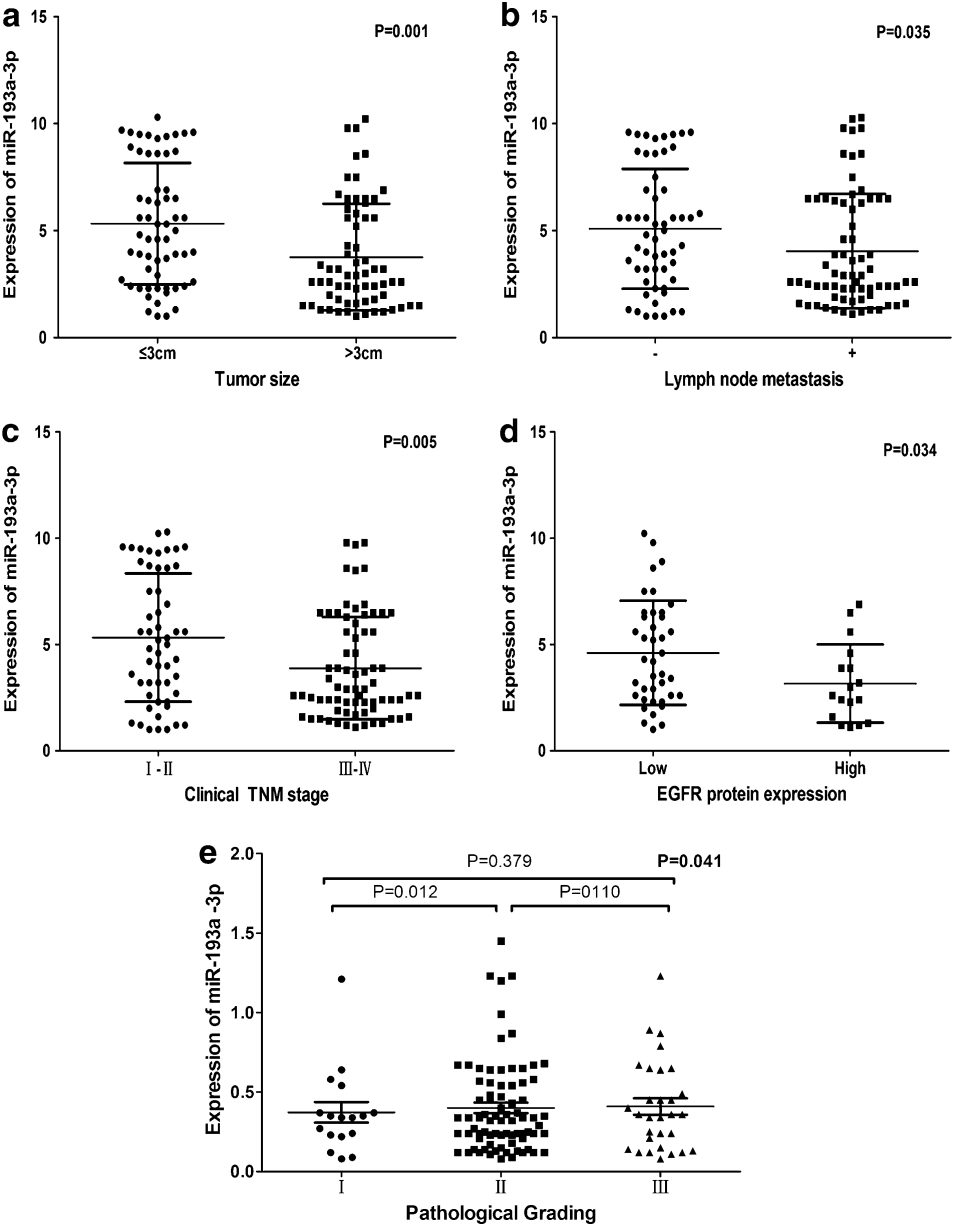
The relationship between miR-193a-3p expression and clinicopathological parameters of NSCLC. **a** Tumor size, **b** Lymph node metastasis, **c** clinical TNM stage, **d** EGFR protein expression, **e** Pathological grading. *P < 0.05, **P < 0.01, ***P < 0.001. The data was representative of two independent experiments. *Error bars* represent SD. *P < 0.05, **P < 0.01, ***P < 0.001 by Student’s *t* test (**a**–**e**).

The associations between miR-193a-3p and patient parameters were summarized in Table 1. Compared with those whose tumor size was >3 cm (3.7574 ± 2.4984), the expression level of miR-193a-3p was markedly down-regulated in those with smaller tumor (5.3203 ± 2.8395, P = 0.001, Fig. 2a). Decreased expression of miR-193a-3p was found in patients with lymph node metastasis (4.0381 ± 2.6739) compared with those without lymph node metastasis(5.0861 ± 2.8002, P = 0.035, Fig. 2b). Besides, in comparison with NSCLC patients in aggressive stages (III and IV, 3.8845 ± 2.4074), the corresponding expression of miR-193a-3p was higher than those in early stages (I and II, 5.3269 ± 3.0154, P = 0.005, Fig. 2c). Moreover, miR-193a-3p expression was remarkably decreased in NSCLC patients with high expression of epidermal growth factor receptor (EGFR) protein (3.1588 ± 1.8426), when compared with those with low EGFR protein expression (4.6058 ± 2.4525, P = 0.034, Fig. 2d). To identify whether there existed differences of miR-193a-3p expression in different pathological grading, we performed ANOVA test. As a consequence, significant difference was identified among different grades in relevant with expression of miR-193a-3p (F = 3.271, P = 0.041, Fig. 2e) and miR-193a-3p expression was much lower in grading I (3.0353 ± 1.9371) compared to grading II (4.8827 ± 2.7938, P = 0.012). Simultaneously, Spearman correlation test was conducted to confirm the association of miR-193a-3p expression and clinicopathological parameters. Negative correlations were found between miR-193a-3p and tumor size (r = −0.277, P = 0.002), lymph node metastasis (LNM) (r = −0.186, P = 0.038), TNM (r = −0.226, P = 0.011), EGFR protein expression (r = −0.272, P = 0.041). No statistically significant associations were detected between the expression of miR-193a-3p and the following factors: age, gender, smoking status, vascular invasion, EGFR mutation, EGFR amplification or histological classification (Table 1).

Among the 57 patients followed-up, 28 patients had low miR-193a-3p expression, and 29 had high expression. Univariate analysis of overall survival (OS) showed no significant relationship with the expression level of miR-193a-3p (data not shown, P = 0.614).

### Overexpression of AEG-1 in NSCLC FFPE tissues and its correlation with the clinical pathological parameters

**Fig. 3:**
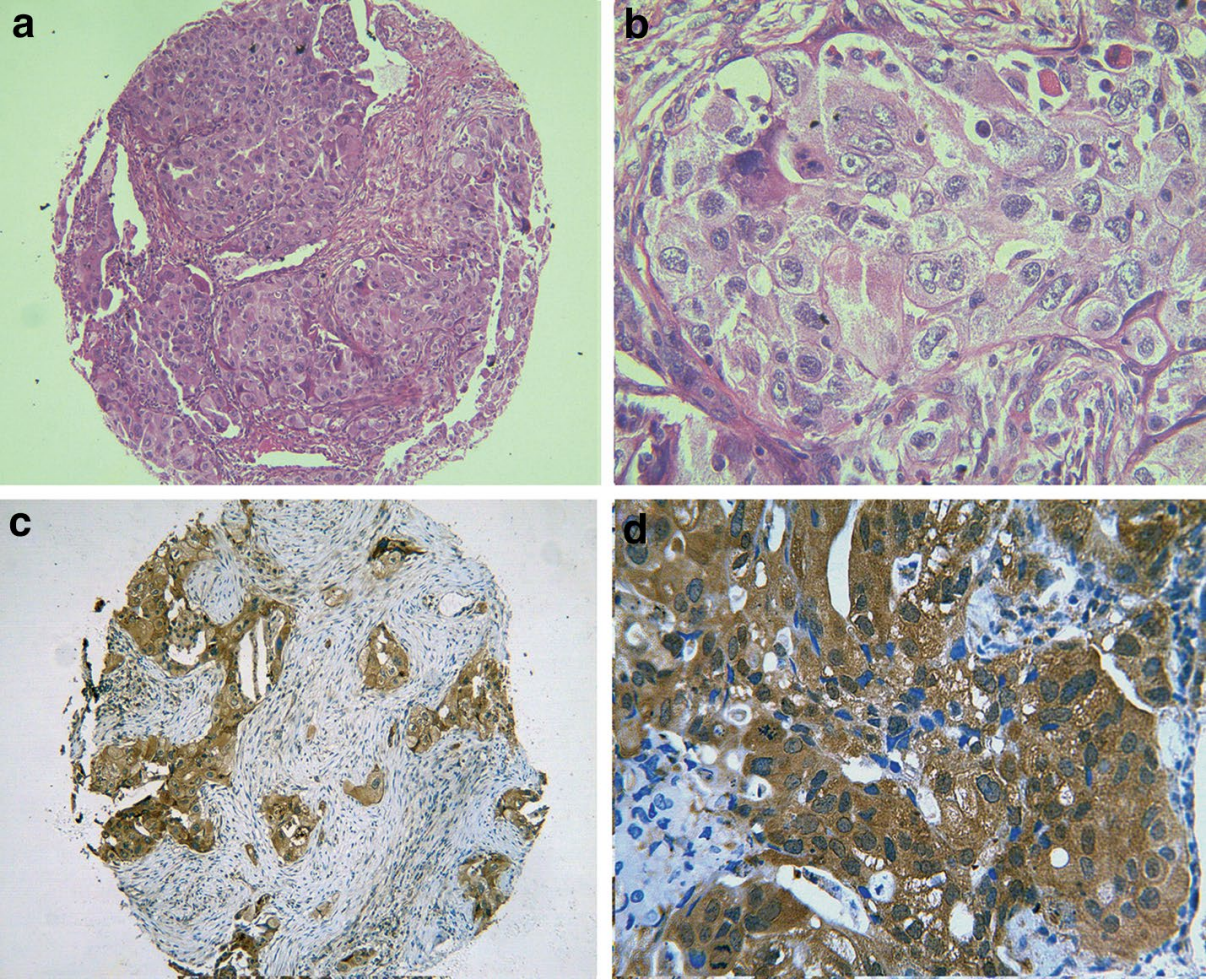
Hematoxylin/eosin and immunohistochemical staining of astrocyte elevated gene-1(AEG-1) in lung adenocarcinoma. Hematoxylin/eosin (HE) staining of lung adenocarcinoma tissues with AEG-1 expression (**a**, ×100; **b**, ×400) and immunohistochemical staining for AEG-1 in lung adenocarcinoma (**c**, ×100; **d**, ×400)

**Fig. 4:**
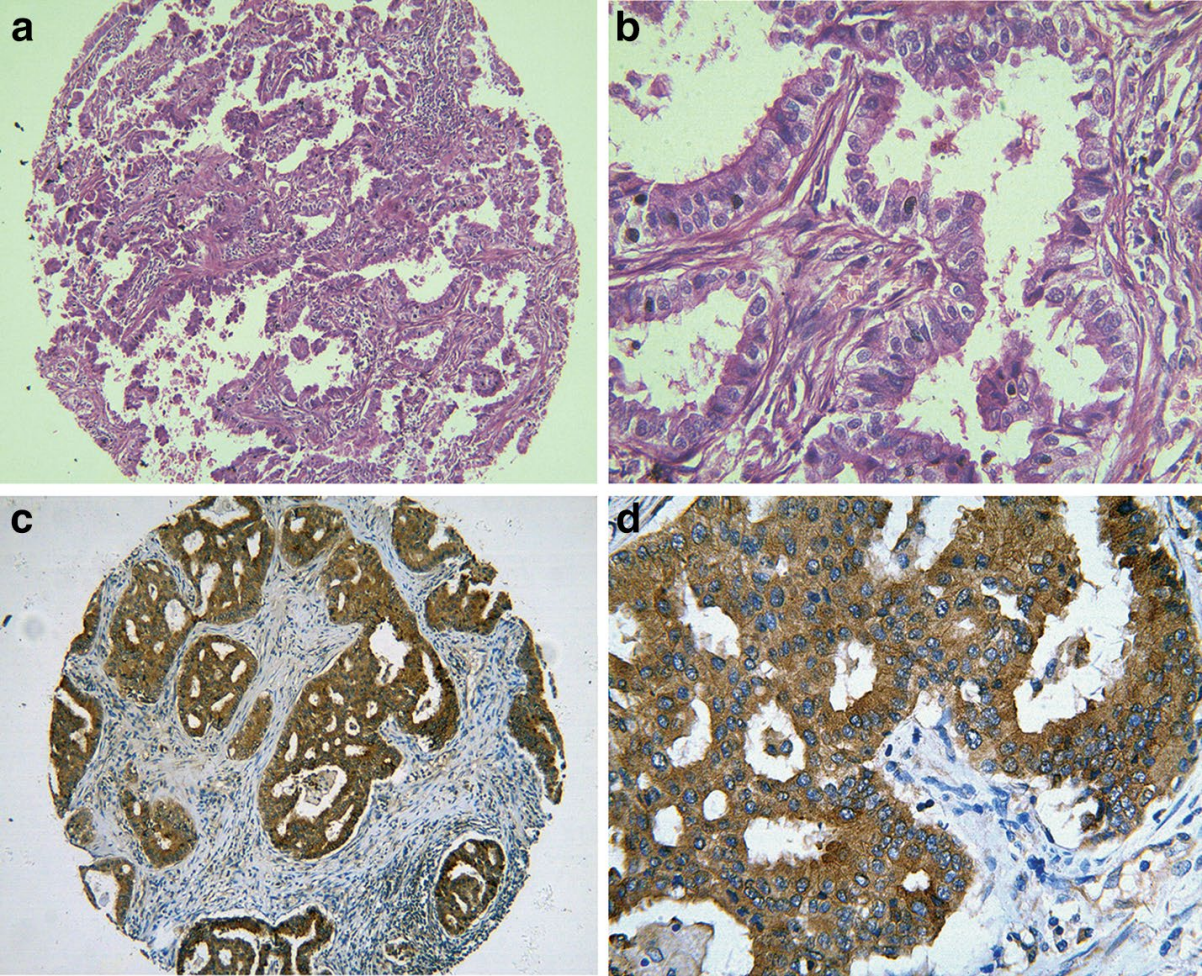
Hematoxylin/eosin and immunohistochemical staining of astrocyte elevated gene-1(AEG-1) in lung squamous carcinoma. Hematoxylin/eosin (HE) staining of lung squamous carcinoma tissues with AEG-1 expression (**a**, ×100; **b**, ×400) and strong cytoplasmic staining for AEG-1 in lung squamous carcinoma (**c**, ×100; **d**, ×400).

To further examine whether the expression of AEG-1 protein was related to the clinical progression of NSCLC, we examined 339 paraffin-embedded NSCLC tissues and 30 normal lung tissues with immunohistochemical staining. As shown in Table 2, AEG-1 protein expression was detected in 172 of 339 (50.7%) NSCLC, which was significantly higher than that of normal lung tissues (23.3%, P = 0.004). Immunohistochemical staining of the AEG-1 protein was shown in Figs. 3, 4. The diagnostic value of AEG-1 as a biomarker in lung cancer was analyzed with ROC curve. The AUC was 0.637 (95% CI 0.540–0.734, P = 0.013), while the sensitivity and specificity were 0.507 and 0.767, respectively. Meanwhile, expression of AEG-1 protein was remarkably increased in NSCLC with larger size (Z = 4.414, P < 0.001), advanced clinical TNM stages (Z = 3.019, P = 0.003) and LNM status (Z = 4.274, P < 0.001). Moreover, AEG-1 protein expression was upregulated in the following NSCLC subgroups:cinar adenocarcinoma (P = 0.014), papillary adenocarcinoma (P = 0.002) and broncholoalveolar cell carcinoma (P = 0.001). Besides, the relationships between AEG-1 expression and clinicopathological features were further confirmed by Spearman correlation test. The results identified that expression of AEG-1 was positively relative to tumor size (r = 0.240, P < 0.001), TNM stages (r = 0.164, P = 0.002) and LNM (r = 0.232, P < 0.001). These consequences demonstrated that AEG-1 expression was significantly correlated with the development of NSCLC.

**Table 2:** The relationship of AEG1 and other clinicopathological parameters in NSCLC

NSCLC	Number	AEG1 negative (n, %)	AEG1 positive (n, %)	χ^2^	P
Tissues				−2.875	0.004
Normal tissues	30	23 (76.7)	7 (23.3)		
NSCLC	339	167 (49.3)	172 (50.7)		
Gender				−0.969	0.332
Male	254	129 (50.8)	125 (49.2)		
Female	85	38 (44.7)	47 (55.3)		
Age (years)				−0.616	0.538
<60	181	92 (50.8)	89 (49.2)		
≥60	158	75 (47.5)	83 (52.5)		
Tumor size				−4.414	<0.001
>7	44	8 (18.2)	36 (81.8)		
≤7	295	159 (53.4)	136 (46.1)		
TNM				−3.019	0.003
I–II	286	151 (52.8)	135 (47.2)		
III–IV	53	16 (30.2)	37 (69.8)		
LNM				−4.274	<0.001
Yes	115	38 (33.0)	77 (67)		
No	224	129 (57.6)	95 (42.4)		
Distal metastasis				−1.474	0.140
Absent	323	162 (50.2)	161 (49.8)		
Present	16	5 (31.3)	11 (68.7)		
Pathological grading				0.648^a^	0.723
I	39	20 (51.3)	19 (48.7)		
II	92	51 (55.4)	41 (44.6)		
III	130	65 (50)	65 (50)		
Histology				3.090	0.543
	127	59 (46.5)	68 (53.5)		
	175	92 (52.6)	83 (47.4)		
	28	12 (42.9)	16 (57.1)		
	8	3 (37.5)	5 (62.5)		
	1	1 (100)	0 (0.00)		
Adenocarcinoma classification				9.054	0.029
Acinar adenocarcinoma	83	42 (50.6)	41 (49.4)	−2.464	0.014
Broncholoalveolar	18	5 (27.8)	13 (72.2)	−3.291	0.001
Papillary adenocarcinoma	19	6 (31.6)	13 (68.4)	−3.097	0.002
Mucinous carcinoma	7	6 (85.7)	1 (14.3)	−0.516	0.719

Pathological grading I vs. II: Z = −0.435, P = 0.664; I vs. III: Z = −0.142, P = 0.889; II vs. III: Z = −0.797, P = 0.426.

*NSCLC* non-small-cell lung cancer, *AEG-1* astrocyte elevated gene-1, *TNM* tumor node metastasis, *LNM* lymph node metastasis.

^a^Kruskal–Wallis H test was performed.

### The potential relationship between miR-193a-3p and AEG-1

**Fig. 5:**
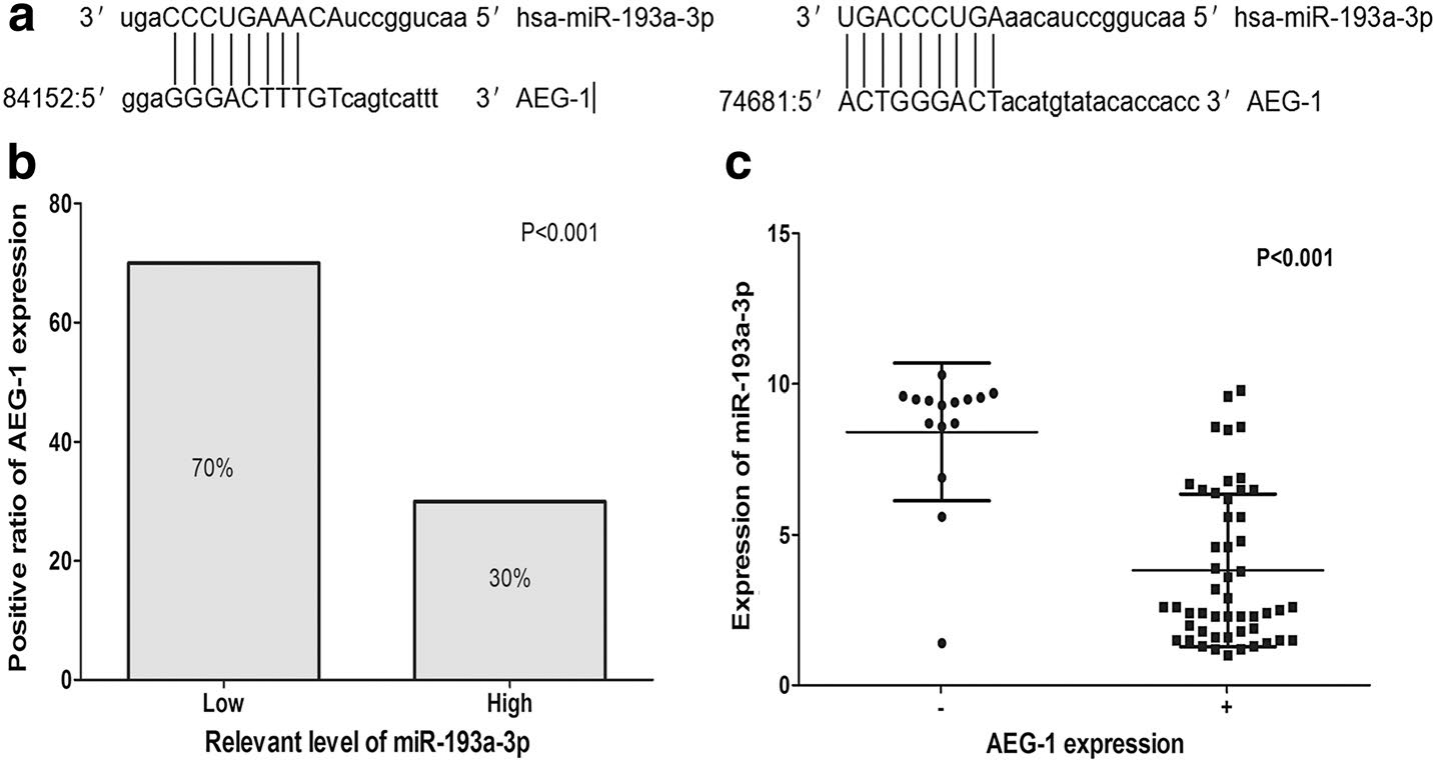
AEG-1 is a potential target gene of miR-193a-3p and miR-193a-3p was negatively correlated with AEG-1 in NSCLC. **a** MiRecords predicted two different binding sites with AEG-1 3′-UTR region. **b** Inverse correlation of AEG-1 protein levels detected by IHC in tissues expressing low and high levels of miR-193a-3p. **c** qRT-PCR of miR-193a-3p expression levels determined in 65 NSCLC individuals with classified into low and high AEG-1 expression. *Error bars* represent standard deviation (SD). The statistical analysis was performed using Student’s t test (**b**, **c**).

To identify the potential target of miR-193a-3p, miRBase, PicTar, TargetScan, Tarbase, miRanda and miRecords (http://mirecords.biolead.org/) bioinformatics methods were used. AEG-1 had the complementary sequences to miR-193a-3p (Fig. 5a). We then detected the levels of miR-193a-3p and AEG-1 at the same time in cohort III and investigated their correlation. When we divided the patients into high miR-193a-3p and low miR-193a-3p groups, AEG-1 positive expression accounted for 70% in the group with decreased expression of miR-193a-3p (Fig. 5b). We further compared the difference of miR-193a-3p level according to the expression of AEG-1. We observed that miR-193a-3p was upregulated in AEG-1 negative patients (8.4147 ± 2.2795) comparing with those with AEG-1 positive expression (3.8200 ± 2.5370, P < 0.001, Fig. 5c). Lastly, correlation analysis also demonstrated that level of miR-193a-3p was inversely associated with the expression of AEG-1 in 65 cases of NSCLC (r = −0.564, P < 0.001).

## Discussion

MiRNAs are widely confirmed as significant factors in regulating the progression of malignant tumors. Among all of these miRNAs, down-regulation of miR-193a-3p was closely relevant to the proliferation, apoptosis, metastasis, invasion and prognosis of a certain type of cancers, especially in NSCLC. MiR-193a-3p was demonstrated to be specifically methylated in NSCLC patients, in contrast, methylation indeed contributed to the decreased expression of miR-193a-3p in patients with NSCLC [14, 16]. Wang et al. [16] reported that methylation-silencing of miR-193a-3p was in favor of promoting proliferation and inhibiting apoptosis through repressing of NF-κB and MCL1. MiR-193a-3p methylation was also found to be more frequent in adenocarcinomas, a subtype of NSCLC, while no evidence testified the association between miR-193a-3p and worse overall survival (OS) or disease free survival (DFS) in patients with NSCLC [14]. Moreover, another two reports identified that miR-193a-3p suppressed the aggressiveness of NSCLC by negatively regulating ERBB4 in vivo and in vitro [15, 17]. The study reported by Liang et al. verified that the restoration of miR-193a-3p in NSCLC repressed the expression of ERBB4 and promoted apoptosis and then inhibited proliferation and invasion [15]. Additionally, decreased expression of miR-193a-3p/5p was remarkably in relation to the TNM and LNM in NSCLC by targeting ERBB4, S6K2, PIK3R3 and mTOR [17]. Besides, metastasis is thought to be the main factor for the mortality of lung cancer patients, which may provide novel insights for preventing the aggression of NSCLC [27].

In our study, down-regulation of miR-193a-3p was confirmed in NSCLC tissues compared with matching normal tissues. The ROC curve also demonstrated that miR-193a-3p expression could act as a novel biomarker for diagnosis of NSCLC. Furthermore, the results identified that overexpression of miR-193a-3p was inversely related to the tumor size, which was consistent with the results proved by Wang et al. [16] and Liang et al. [15]. In addition, according to the American Joint Committee on Cancer (AJCC) staging system for lung cancer, tumor diameter is essential for early stage NSCLC [27]. Compared with their corresponding groups, miR-193a-3p expression was much lower in NSCLC patients with lymph node metastasis and advanced TNM stages, in agreement with the previous studies [17]. Also, deregulation of miR-193a-3p was discovered to be significantly associated with EGFR protein expression and pathological grading. However, no evidence proved the relationship between expression of miR-193a-3p and OS. Thus, our current study might provide more information on understanding the inhibition of miR-193a-3p on tumorigenesis and aggression in NSCLC patients.

Numerous researches confirmed that increased expression of AEG-1 played crucial roles in the carcinogenesis of NSCLC [19–24]. As reported, overexpression of AEG-1 could promote NSCLC metastasis and invasion and repress apoptosis by activating the PI3K/Akt pathway and enhancing the level of matrix metalloproteinase-9 (MMP-9) and anti-apoptotic protein Bcl-2 [19, 22, 23]. In addition, suppression of AEG-1 by UA (Ursolic acid)/miR-30a/miR-145 may be able to prevent metastasis and invasion of lung cancer through inhibiting epithelial–mesenchymal transition (EMT) in vitro [20, 21, 24]. On the opposite, there was also another study investigating that up-regulation of AEG-1 protein was in favor of predicting better OS and suppressing proliferation, metastasis [28]. In the current study, when compared with the normal lung tissues, AEG-1 protein expression was notably increased in NSCLC tissues. There existed a positive association between AEG-1 expression and tumor size, clinical TNM stages and LNM. Accordant to previous studies, the expression of AEG-1 protein could accelerate the processes of NSCLC, including tumor cell proliferation and metastasis. As reported by Liu et al. [20] and Wang et al. [24], AEG-1 could be repressed by the up-regulation of miRNAs in aggressiveness of NSCLC. However, no study has demonstrated the correlation between expression of miR-193a-3p and AEG-1. In current study, it was the first time to find the complementary sequences between AEG-1 and miR-193a-3p. Meanwhile, increased expression of miR-193a-3p was accompanied with the decreased expression of AEG-1 protein. Thus miR-193a-3p might have the potential to contribute to tumor formation and development by partially targeting AEG-1 in patients with NSCLC. However, since in silico prediction has an estimated false discovery rate of 60%, the relationship between AEG-1 and miR-193a-3p needs further investigation.

## Conclusion

In conclusion, our current study indicates the down-expression of miR-193a-3p and overexpression of AEG-1 protein are both involved in the metastasis and progression of NSCLC. Our findings also suggest that AEG-1 could have the potential to be a target gene of miR-193a-3p. Therefore, the miR-193a-3p/AEG-1 interaction might provide a newly effective strategy for NSCLC diagnosis and therapy. However, new in vitro and in vivo tests are required to explore the relationship and mechanism of miR-193a-3p with AEG-1.

## Methods

This research included three independent cohorts of lung cancer patients receiving surgical resection without treatment in the First Affiliated Hospital of Guangxi Medical University from January 2010 to February 2014 (cohortI: n = 125; cohort II: n = 369; cohort III: n = 65). Pathological tissues involved in cohort I were randomly selected from tumor tissues and their paired nonmalignant NSCLC tissues, aged from 23 to 90 years (mean age 61.10 years). Cohort II contained 369 cases including 339 cancer tissues (aged from 11 to 84 years; mean 58.17 years) and 30 normal lung tissues (aged from 19 to 73 years; mean 54.03 years). The clinicopathological information of these two cohorts were independently shown in Tables 1 and 2. In cohort III independent from cohort I and II, all 65 patients were diagnosed as NSCLC. The study was approved by the Ethical Committee of the First Affiliated Hospital of Guangxi Medical University, and informed consent was obtained from the patients for the usage of the tissues for research. All samples were reviewed and diagnosed by two independent pathologists.

RNA isolation and RNA normalization were performed as previously described [29–31]. A combination of miR-191 and miR-103 was the most stable housekeeping genes for detection of miR-193a-3p expression [30, 32]. The commercial kits used in our study were TaqMan^®^ MicroRNA Assays (4427975, Applied Biosystems, Life Technologies Grand Island, NY 14072 USA) and TaqMan^®^ MicroRNA Reverse Transcription Kit (4366596, Applied Biosystems, Life Technologies Grand Island, NY 14072 USA) in a total volume of 10 µL. Sequence of targeted miRNA and reference miRNAs used were as follows: miR-193a-3p (Applied Biosystems Cat. No. 4427975-000492): AACUGGCCUACAAAGUCCCAG; miR-191 (Applied Biosystems Cat. No. 4427975-002299): CAACGGAAUCCCAAAAGCAGCU; miR-103 (Applied Biosystems Cat. No. 4427975-000439): AGCAGCAUUGUACAGGGCUAUGA. Real-time qPCR to detect miRNA relevant level was performed with Applied Biosystems PCR (7900). The formula 2^−Δcq^ was applied to calculate the expression level of miR-193a-3p.

AEG-1 rabbit multi-clonal antibody was purchased from Santa Cruz Biotechnology, Inc, Heidelberg, Germany. The positive intensity and positive ratio of AEG-1 protein was evaluated by two pathologists with no prior knowledge of the clinicopathological outcomes of the patients. The scores of immunopositive cells was semiquantitatively calculated as follows: firstly, scoring according to staining intensity: negative (0), weak (1), moderate (2), strong (3); then, scoring based on the proportion of positive stained cells: 0 (0%), 1 (1–25%), 2 (26–50%), 3 (51–75%), and 4 (76–100%). If the pathological scores of multiplication between staining intensity and the percentage of positive cells were over 2, it was regarded as immunoreaction positive.

Statistical analysis was carried out by using SPSS 20.0. Student’s *t* test and Chi square tests were applied for estimating the difference between two groups. One-way analysis of variance (ANOVA) test and Kruskal–Wallis H test were performed to evaluate the correlation between three or more groups. Receiver operating characteristic (ROC) curve was used to predict NSCLC and non-tumorous tissues by miR-193a-3p expression levels. Besides, the Kaplan–Meier method and the log-rank test were constructed to evaluate the survival analysis, and Spearman correlation was conducted to analyze the correlation of miR-193a-3p and AEG-1 expression level and clinicopathological parameters. Value of P < 0.05 was considered statistically significant.

## Abbreviations

NSCLC: non-small-cell lung cancer
HCC: hepatocellular Carcinoma
miR-193a-3p: microRNA-193a-3p
AEG-1: astrocyte elevated gene-1
MTDH: metadherin
Lyric: lysine-rich CEACAM1
qRT-PCR: quantitative Real-time PCR
TNM: tumor node metastasis
Clinical TNM stage: The most common systems for staging employs the TNM classification. A “T” score is based upon the size and/or extent of invasion. The “N” score indicates the extent of lymph node involvement. The “M” score indicates whether distant metastases are present
LNM: lymph node metastasis
miRNAs: microRNAs
OS: overall survival
EGFR: epithelial growth factor receptor
EMT: epithelial–mesenchymal transition
AJCC: American Joint Committee on Cancer
